# Fetal Movement Counting Improved Identification of Fetal Growth Restriction and Perinatal Outcomes – a Multi-Centre, Randomized, Controlled Trial

**DOI:** 10.1371/journal.pone.0028482

**Published:** 2011-12-21

**Authors:** Eli Saastad, Brita A. Winje, Babill Stray Pedersen, J. Frederik Frøen

**Affiliations:** 1 Faculty of Health, Nutrition and Management, Oslo and Akershus University College of Applied Sciences, Oslo, Norway; 2 Division of Epidemiology, Norwegian Institute of Public Health, Oslo, Norway; 3 Division of Women and Children, Rikshospitalet, Oslo University Hospital, Oslo, Norway; 4 Institute of Clinical Medicine, University of Oslo, Oslo, Norway; Institute of Clinical Effectiveness and Health Policy, Argentina

## Abstract

**Background:**

Fetal movement counting is a method used by the mother to quantify her baby's movements, and may prevent adverse pregnancy outcome by a timely evaluation of fetal health when the woman reports decreased fetal movements. We aimed to assess effects of fetal movement counting on identification of fetal pathology and pregnancy outcome.

**Methodology:**

In a multicentre, randomized, controlled trial, 1076 pregnant women with singleton pregnancies from an unselected population were assigned to either perform fetal movement counting from gestational week 28, or to receive standard antenatal care not including fetal movement counting (controls). Women were recruited from nine Norwegian hospitals during September 2007 through November 2009. Main outcome was a compound measure of fetal pathology and adverse pregnancy outcomes. Analysis was performed by intention-to-treat.

**Principal Findings:**

The frequency of the main outcome was equal in the groups; 63 of 433 (11.6%) in the intervention group, versus 53 of 532 (10.7%) in the control group [RR: 1.1 95% CI 0.7–1.5)]. The growth-restricted fetuses were more often identified prior to birth in the intervention group than in the control group; 20 of 23 fetuses (87.0%) versus 12 of 20 fetuses (60.0%), respectively, [RR: 1.5 (95% CI 1.0–2.1)]. In the intervention group two babies (0.4%) had Apgar scores <4 at 1 minute, versus 12 (2.3%) in the control group [RR: 0.2 (95% CI 0.04–0.7)]. The frequency of consultations for decreased fetal movement was 71 (13.1%) and 57 (10.7%) in the intervention and control groups, respectively [RR: 1.2 (95% CI 0.9–1.7)]. The frequency of interventions was similar in the groups.

**Conclusions:**

Maternal ability to detect clinically important changes in fetal activity seemed to be improved by fetal movement counting; there was an increased identification of fetal growth restriction and improved perinatal outcome, without inducing more consultations or obstetric interventions.

**Trial Registration:**

ClinicalTrials.gov NCT00513942

## Introduction

Maternal perception of a gradual diminishment of fetal activity is a significant marker of a vulnerable fetus and can indicate chronic fetal compromise [1]–[5], precede fetal growth restriction, stillbirth, preterm birth and emergency Caesarean section [6]–[8]. The most important marker of decreased fetal activity is what women perceive as decreasing fetal movements [2], [9]. Maternal vigilance of fetal activity and timely reporting to healthcare providers when experiencing a decrease may prevent perinatal morbidity and mortality [2], [10]–[12]. However, there is only low level evidence on how to counsel women so they are empowered to timely identify and act on decreased fetal movements [3], [10].

Fetal movement counting is a means of screening fetal status. It is developed as a simple, inexpensive and easily accessible tool to support the mother in monitoring her baby's well-being to identify alarming behavior in time to intervene [13], [14]. The fetal movement monitoring methods can be divided roughly into two understandings [15], formal fetal movement counting with specified limits for decreased fetal activity, as opposed to merely raising maternal awareness and vigilance to fetal activity and the significance of decreased fetal movement. The latter approach is consistent with national guidelines in the United Kingdom [16], the US [17] and Norway [18]. Fetal movement counting can be promoted as both, as counting *per se* may serve as an organized daily effort to ensure vigilance to fetal movements, and thus, improve the ongoing maternal self-screening [19].

There is a lack of evidence of the sensitivity and specificity for the variety of quantitative definitions of decreased fetal movement that has been proposed over the years and there is no conclusive evidence that any of them reduce perinatal morbidity and mortality [2], [3], [13], [14], [20]. Formal fetal movement counting is disputed among health professionals [16], [18], [21]–[23]. Critiques argue that fetal movement counting may cause psychological distress [20]–[22] and induce superfluous consultations and obstetric interventions (induction of labor, Caesarean section) [20]. However, we have recently demonstrated in our trial that fetal movement counting is reassuring to mothers, and leads to lower levels of concern [24]. We aimed to assess the effects of increased awareness towards fetal activity by use of a fetal movement counting chart on antenatal identification of fetal pathology, pregnancy outcomes and the frequency of interventions during delivery.

## Methods

The protocol for this trial and supporting CONSORT checklist are available as supporting information; see Checklist S1 and Protocol S1.

### Ethics Statement

Ethical approval was obtained from The Regional Committee for Medical Research Ethics (reference S-07188a) 7 May 2007, and by The Norwegian Data Inspectorate and Directorate for Health (reference 07/2504) 19 July 2007. The study was registered in www.clinicaltrials.gov protocol registration system (number NCT00513942).

### Trial design, setting and subjects

**Figure 1:**
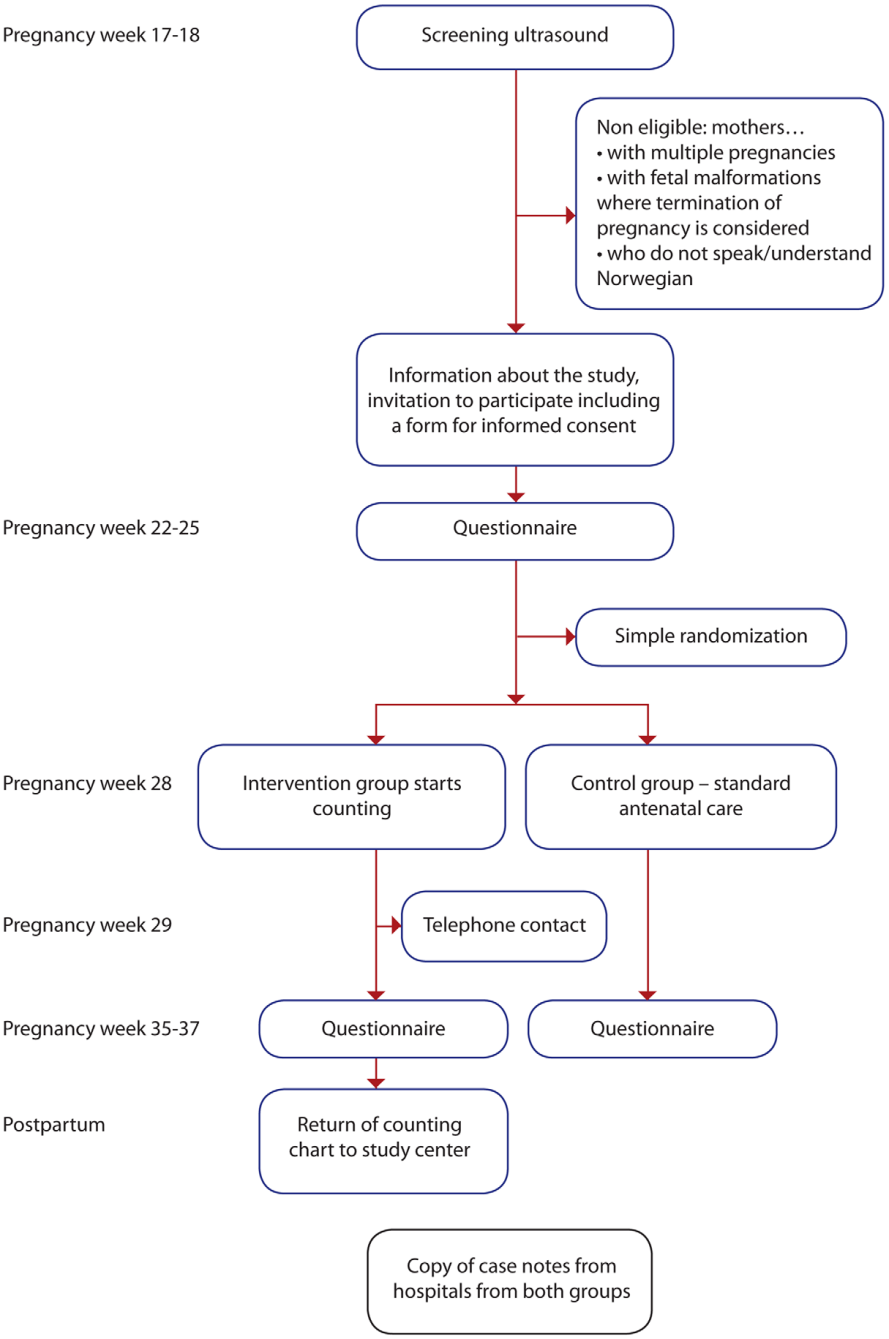
Flow chart data collection.

In a randomized, controlled trial pregnant women were allocated to one of two groups, the intervention group who were instructed to perform fetal movement counting from gestational week 28, and the control group who received standard antenatal care according to the Norwegian guidelines. In Norway, antenatal care is a public health care service free of charge to which almost all pregnant women adhere. Participants were approached during their regular ultrasound screening in pregnancy weeks 17–19 (flow-chart in figure 1). The recruitment brochure provided information about the purpose of the study: to improve our knowledge about the effects of fetal movement counting on expectant mothers. Eligible women were Norwegian-speaking women with singleton pregnancies; excluding pregnancies where severe anomalies or other causes for considering termination of the pregnancy were identified. Women were recruited from September 2007 through November 2009 at nine Norwegian hospitals from both urban and rural populations, handling total of 8200 births annually.

**Figure 2:**
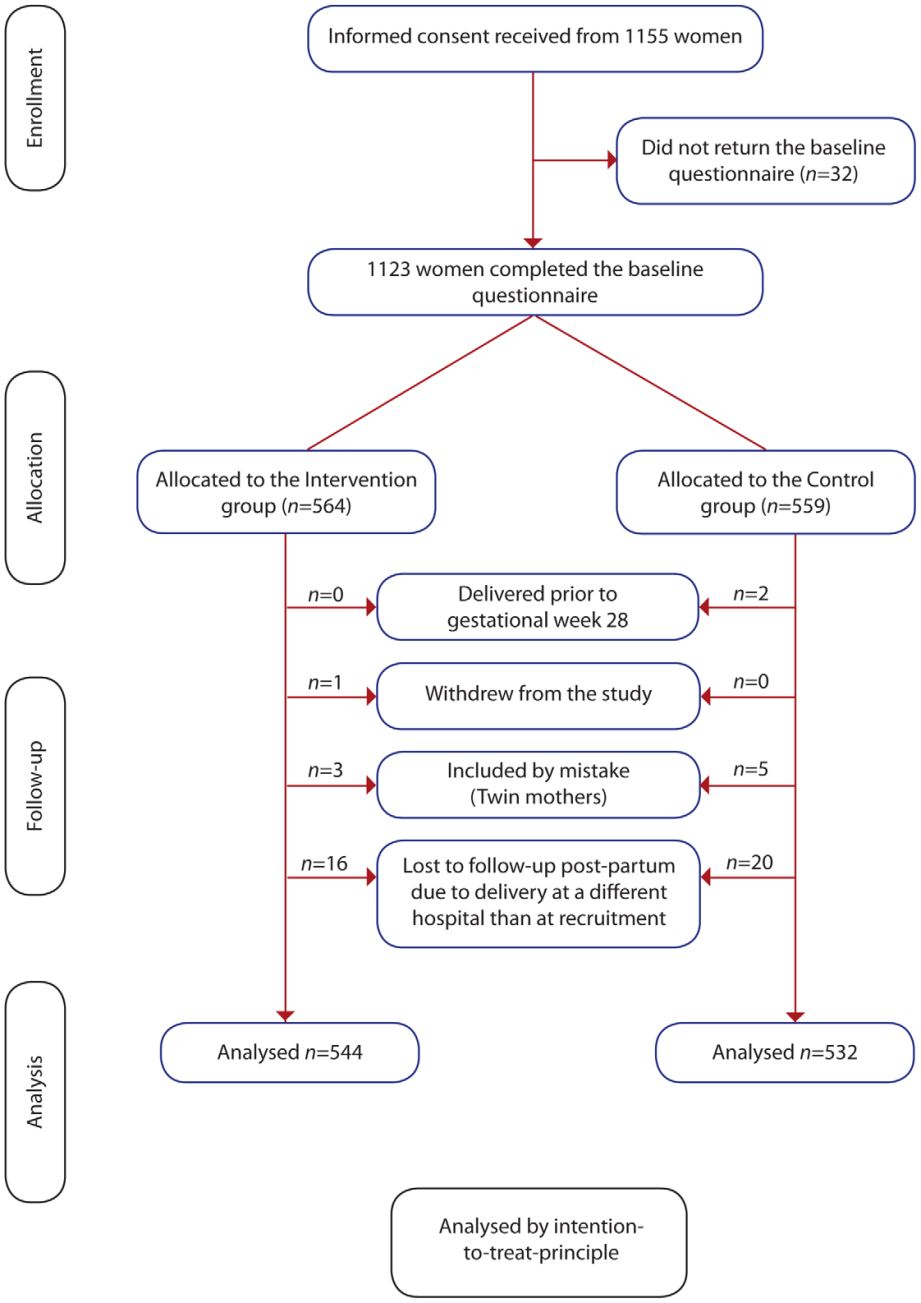
Flow chart study participants.

The current study was a part of a more comprehensive evaluation of fetal movement counting. The two other studies required a completed questionnaire in pregnancy week 22 for participants to be eligible for allocation [24], [25]. Thirty six women (3.4%) were lost to follow up due to delivery at a different hospital than where they were recruited (figure 2). Among these, there was one stillbirth caused by a lethal malformation.

Demographic and obstetric information was obtained from case notes received from the hospitals after the delivery. Registration of these data was blinded for allocation. Data on maternal age, parity, marital status and smoking habits in the total population of women who gave birth were obtained from the Medical Birth Registry of Norway [26]. The study sample was representative for the total population of pregnant women in Norway with respect to parity and the proportion of women of age ≥35 years, but there was a lower proportion of smoking women in the study sample than in the total population (data not shown).

### Randomization

Simple randomization was determined according to a computer-generated random allocation list with an equal probability of ending up in each of the groups. The allocation sequence was concealed until participants were assigned to trial groups. After allocation, blinding for group assignment was not desirable neither for the participants nor their care providers, as use of a fetal movement counting chart was intended to be an active tool for interaction between the woman and her midwife or physician.

### The intervention

**Figure 3:**
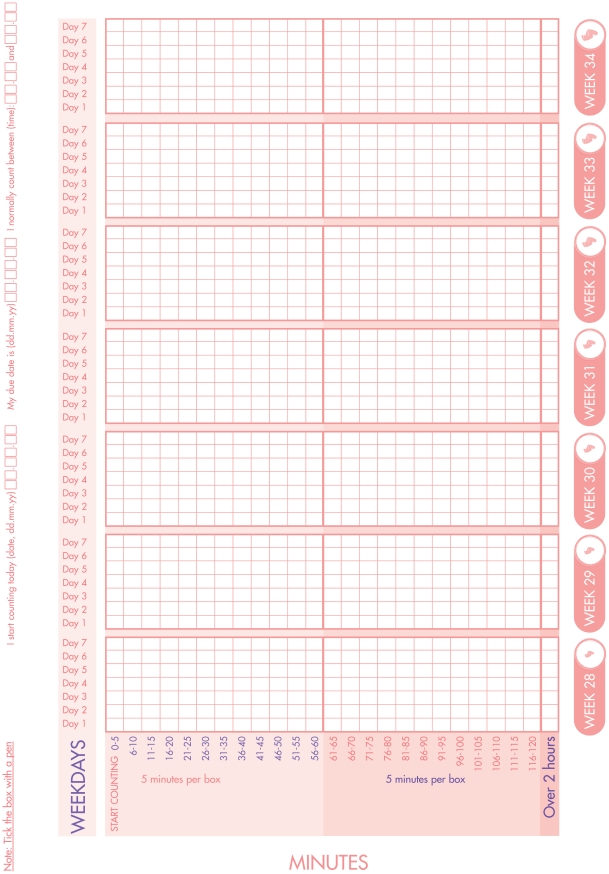
The fetal movement counting chart (the first of two pages).

Women in the intervention group received an information brochure, including instructions on how to use a fetal movement chart, and were asked to count fetal movements daily from gestational week 28. A modified *Count-to-ten* method [27] (figure 3), previously tested in a Norwegian population [11], [12], [28] was used. Further assessment of the methods for fetal movement counting has been presented elsewhere [3], [15], [29]. A midwife or an obstetrician from the participating hospitals or the research study group called women in the intervention group within two weeks after counting-start to support them in the interpretation of the counting method. Women were informed that their subjective assessment of a significant and sustained reduction in normal fetal activity for the baby was the primary marker of decreased fetal activity, i.e. their perception of a change – taking priority over any formal alarm limits for decreased fetal activity.

### Instruments and measures

Primary outcome was a compound measure of the following: (i) fetal growth restriction <2.5^th^ centile; (ii): emergency Caesarean section on fetal indication; (iii) oligohydramnios (as defined by the clinicians); (iv) pathological blood flow in arteria umbilicalis; (v) maternal perception of absent fetal movements for more than 24 hours before admission to hospital, or (vi) perinatal death. Secondary outcomes were (i) Apgar scores <4 at 1 and 5 minutes; (ii) fetal growth restriction <2.5^th^ centile unidentified prior to birth; (iii) the total number of consultations for decreased fetal activity; (iv) use of health resources in evaluation of these pregnancies, and (v) interventions prior to or during delivery. Fetal growth restriction was defined as a birth weight <2.5^th^ centile adjusted for maternal height and weight in early pregnancy, and baby's sex [30], [31], or an antenatal ultrasound measure estimating fetal growth <2.5^th^ percentile birth weight (i.e. <21.5% negative deviation), or a negative trend on serial antenatal ultrasounds.

Maternal characteristics included demographic information and risk factors according to Norwegian Guidelines [32], including: (i) *general information*: age, educational level, marital status, Body Mass Index, nicotine and alcohol consumption, and country of origin; (ii) *obstetric risk factors:* previous pregnancy with fetal growth restriction, stillbirth >21 weeks of gestation, preterm delivery, serious preeclampsia or malformations; (iii) *pre pregnancy risk factors*: hypertension, chronic renal or coronary disease, known diabetes type I or II, inflammatory and rheumatoid diseases, coagulopathy, epilepsy or hypothyreosis, and (iv) *maternal complications identified during pregnancy*: hypertension, preeclampsia, preterm contractions, prolonged preterm rupture of membranes, haemorrhage >27 weeks of pregnancy, diabetes (any type), urinary tract infection or other relevant infections.

As an indicator of the user-friendliness of the counting chart, compliance was measured. A total of 427 (78.5%) women returned the chart. Of these, 331 women (77.5%) completed the counting chart more than 50 per cent of the days during the period and at least two days each week. Compliance did not vary between any subgroups.

### Sample size calculation

Sample size calculation was made using the computer program PS Power and Sample Sizes [33]. The effect size was estimated by means with standard deviations (SD) for continuous variables. The goal was to increase the identification of fetal pathology as a predictor for need of examination or intervention. This was measured by rates of identification of risk according to the original compound measure. Expected prevalence was 13.5%, estimated from results in previous studies in our population [34] or the Medical Birth registry of Norway [26]. Detectable changes were estimated to be a 10% increase of identification of these risk pregnancies, which gave an estimated sample of 538 in each arm of the trial with 80% power and a significance level of 0.05.

### Statistical analyses

The data were analyzed using SPSS 17.0 (SPSS Inc., Chicago, IL) and Episheet [35]. Demographic and clinical characteristics were summarized by the mean ± one SD for continuous variables and as frequency counts (percentages) for categorical variables. Effect size was analyzed using chi square and Fisher exact tests and included Relative Risk (RR) with its 95% confidence intervals (CI). Comparisons of maternal age, parity, marital status and smoking habits between the study sample and the total population of women delivering in Norway were performed using chi square test.

The significance level was set at p = 0.05. All analyses were performed according to the intention-to-treat principle. Analyses were performed by the researchers without blinding for group assignment. This manuscript is in compliance with the CONSORT statement for reporting trials [36].

## Results

We obtained written informed consent from 1155 women. As demonstrated in the study profile (figure 2), 1076 women were included in the analyses. At randomization, no differences were found between the groups with regard to demographic and clinical characteristics (table 1).

**Table 1:** Baseline demographic and clinical data, N = 1076.

Maternal characteristics	Intervention group, n = 544	Control group, n = 532
	n (%^a^)	n (%^a^)
Maternal age ≥35 yrs	98 (18.0)	106 (19.9)
Primiparous	228 (41.9)	248 (46.6)
Maternal obesity (Body Mass Index ≥30 kg/m^2^)	68 (12.5)	57 (10.7)
≥High school graduate	324 (64.5)	304 (61.8)
Single living	37 (6.8)	31 (5.8)
Daily/occasionally smoking 1^st^ trimester	46 (8.5)	51 (9.6)
Daily/occasionally use of alcohol 1^st^ trimester	36 (7.1)	27 (5.6)
Women of Non-Western origin	25 (4.6)	17 (3.2)
Obstetric risk factors^b^	18 (3.3)	14 (2.6)
Pre-pregnancy risk factors^c^	41 (7.5)	38 (7.1)
Maternal complications during pregnancy^d^	133 (24.4)	131 (24.6)

a Denominators vary due to missing values.

b Previous fetal growth restriction, stillbirth >21 weeks of gestation, preterm delivery, serious preeclampsia or malformations.

c Hypertension, chronic renal or coronary disease, known diabetes type I or II, inflammatory and rheumatoid diseases, coagulopathy, epilepsy or hypothyroidism.

d Hypertension, preeclampsia, preterm contractions, prolonged preterm rupture of membranes, haemorrhage >27^th^ gestational week, diabetes (any type), urinary tract infection, systemic infection or other infection.

The frequency of the primary outcome was equal between the groups; 63 of 433 (11.6%) in the intervention group, versus 53 of 532 (10.7%) in the control group [RR: 1.1 95% CI 0.7–1.5) (p = 0.652)]. The proportion growth-restricted fetuses was similar between the groups (table 2), but these fetuses were more often identified prior to birth in the intervention group than in the control group; 20 of 23 fetuses (87.0%) versus 12 of 20 fetuses (60.0%) in the groups, respectively, [RR: 1.5 (95% CI 1.0–2.1) (p = 0.046)]. There were less adverse outcomes in the intervention group than in the control group (table 2 and table 3). There were no fetal deaths.

**Table 2:** Fetal pathology, clinical management and neonatal outcomes, N = 1076.

	Intervention group	Control group		
	n/N (%)	n/N (%)	RR (95% CI)^a^	P
***FETAL PATHOLOGY***				
Fetal growth restriction^b^	23/543 (4.2)	20/530 (3.8)	1.1 (0.6–2.0)	0.700^c^
Oligohydramnios	16/544 (2.9)	9/532 (1.7)	1.7 (0.8–3.9)	0.174^c^
Malformations	1/544 (0.2)	0	-	1.000^d^
Perinatal death	0/544 (0)	0/532 (0)	-	-
***DELIVERY***				
**Start**				
Spontaneous start	431/544 (79.2)	418/532 (78.6)	1.0 (1.0–1.1)	0.792^c^
Induced vaginal delivery	77/544 (14.2)	76/532 (14.3)	1.0 (0.7–1.3)	0.951^c^
Elective Caesarean section	29/544 (5.3)	34/532 (6.4)	0.8 (0.5–1.4)	0.459^c^
Emergency Caesarean section	7/544 (1.3)	4/532 (0.8)	1.7 (0.5–5.8)	0.383^c^
Inductions or interventions on fetal indication	95/544 (17.5)	90/532 (16.9)	1.0 (0.8–1.3)	0.812^c^
**Intra partum interventions after a spontaneousor induced vaginal delivery**				
Assisted vaginal delivery	34/508 (6.7)	32/494 (6.5)	1.0 (0.7–1.7)	0.891^c^
Emergency Caesarean section	36/508 (7.1)	32/494 (6.5)	1.2 (0.7–1.7)	0.702^c^
***NEONATAL OUTCOME***				
Apgar <4 at 1 minutes	2/544 (0.4)	12/532 (2.3)	0.2 (0.04–0.7)	0.006^d^
Apgar <4 at 5 minutes	0/544 (0)	2/534 (0.4)	-	0.244^d^
Birth weight in grams, mean (SD^f^)	3637 (517)	3611 (499)		0.425^e^
Small for gestational age <2.5^th^ centile^g^	8/543 (1.5)	11/530 (2.1)	0.7 (0.3–1.8)	0.455^c^
Small for gestational age <10^th^ centile^g^	46/543 (8.5)	46/530 (8.7)	1.0 (0.7–1.4)	0.903^c^
Gestational age at birth in days, mean (SD^f^)	280 (10.9)	279 (11.2)		0.321^e^
Preterm delivery	20/544 (3.7)	24/532 (4.5)	0.8 (0.5–1.5)	0.489^c^
Transferred to neonatal care unit^h^	33/544 (6.1)	30/532 (5.6)	1.1 (0.7–1.7)	0.765^c^
Female fetal gender	272/544 (50.0)	275/532 (51.7)	1.0 (0.9–1.1)	0.579^c^

a Relative risk (95% Confidence Interval).

b A baby with an adjusted birth weight below 2.5^th^ percentile, or one ultrasound measurement <−21.5% (2.5^th^ centile), or at least two ultrasound measurements showing a negative growth trend from at least 10% to at least 13.5% negative deviation.

c *P*-values refer to chi square test between the control and intervention groups.

d *P*-values refer to Fisher test between the control and intervention groups.

e *P*-values refer to *T-*test between the control and intervention groups.

f Standard Deviation.

g Birth weight for gestational percentiles, adjusted for maternal height and weight in early pregnancy and baby's sex.

h Admission to neonatal care unit due to reasons with association to growth restriction or fetal distress.

**Table 3:** Consultations for decreased fetal movements, identification of pathology, clinical management and interventions, n = 127.

	Intervention group, n = 71	Control group, n = 56		
	n/N (%)	n/N (%)	RR (95% CI)^a^	P^b^
***IDENTIFICATION OF PATHOLOGY***				
Fetal weight estimate <−10% by ultrasound measurement	8/70 (11.4)	1/54 (1.9)	6.2 (0.8–47.9)	0.042
Fetal distress	7/70 (10.0)	5/55 (9.1)	1.1 (0.4–3.3)	0.864
Oligohydramnios	6/70 (8.6)	2/54 (3.7)	2.3 (0.5–11.0)	0.274
Pathological blood flow in arteria umbilicalis	3/70 (4.3)	0	-	0.124
Other pathology	2/70 (2.9)	3/54 (5.6)	0.5 (0.1–3.0)	0.449
***RESOURCES USED IN EVALUATION OF THESE PREGNANCIES***				
Cardiotocography for a non-stress test	66/69 (95.7)	50/55 (90.9)	1.1 (1.0–1.2)	0.285
Ultrasound for measurement of fetal growth, amnioticfluid or fetal activity	54/69 (78.3)	43/55 (78.2)	1.0 (0.8–1.2)	0.992
Measurement of blood flow in arteria uterina by Doppler	29/66 (43.9)	28/53 (52.8)	0.8 (0.6–1.2)	0.335
***ANY FOLLOW-UP AFTER THE CONSULTATION*** c	33/67 (49.3)	18/56 (32.1)	1.5 (1.0–2.4)	0.055
Recurrent consultation	18/66 (27.3)	10/56 (17.9)	1.5 (0.8–3.0)	0.218
Admission delivery unit for observation	11/67 (16.4)	5/56 (8.9)	1.8 (0.7–5.0)	0.219
Admission delivery unit for induction	4/67 (6.0)	0	-	0.063
Admission delivery unit for emergency Caesarean section	4/67 (6.0)	3/56 (5.4)	1.1 (0.3–4.8)	0.884
***DELIVERY – INTERVENTIONS ON FETAL INDICATIONS***				
Induced start of delivery	14/71 (19.7)	13/56 (23.2)	0.9 (0.4–1.7)	0.633
Emergency Caesarean section	10/71 (14.1)	6/56 (10.7)	1.3 (0.5–3.4)	0.570
Interventions during delivery on fetal indication	17/71 (23.9)	14/56 (25.0)	1.0 (0.5–1.8)	0.891

a Relative risk (95% Confidence Interval).

b P-values refer to chi square tests between the control and intervention groups, respectively.

c Any follow-up after the consultation; recurrent consultation, admission delivery unit for observation, induction or emergency Caesarean section.

The frequency of consultations because of maternal concern for decreased fetal movements did not differ between the groups, 71 of 542 pregnancies (13.1%) in the intervention group, versus 57 of 532 pregnancies (10.7%) in the control group [RR: 1.2 (95% CI 0.9–1.7) (p = 0.228)]. The mean gestational age at the time of maternal report was similar between the groups: mean gestational day (SD) was 254 (range 196–295) (SD 27.6) and 258 (range 198–296) (SD 25.9) in the groups, respectively (p = 0.402). Among the consultations for decreased fetal movements, more often a fetus with a <−10% weight estimate was identified in the intervention versus the control group (Table 3).

There were no statistically significant differences between the groups regarding the frequency of interventions prior to or during delivery, neither in the total sample (table 2) nor among women presenting with decreased fetal activity (table 3). Indication for induction in the 153 vaginal deliveries did not vary between the intervention and control groups, with the following proportions in the groups, respectively: 48.1% vs. 53.3% were induced due to post-term pregnancy, 36.4% vs. 36.9% were based on fetal indication and 15.6% vs. 9.5% were based on maternal indication (p = 0.173).

## Discussion

This study has suggested that an intervention involving fetal movement counting, compared to no intervention, was associated with improved identification of fetal growth restriction and a reduction in fetuses with severely low Apgar scores – both known to be associated with further adverse neonatal and childhood outcomes [37]–[40]. Maternal report of decreased fetal movement did not increase in the intervention group, nor did the frequency of interventions prior to or during delivery. The main outcome measure was similar in both groups. However, further deliberations after publishing the original protocol concluded that this compound measure does not reflect the intended primary outcomes, and is not amenable to meaningful interpretation. There are several reasons for this. Firstly, there is currently no cure for fetal growth restriction, and thus no screening can affect the incidence. Secondly, the clinical decisions underlying emergency caesarean are ambiguous and can equally be seen as the result of successful screening as an adverse outcome. Similarly, the diagnoses of oligohydramnios and pathological blood flow in arteria umbilicalis are ambiguous for successful screening or adverse outcome. Finally, maternal behavior associated with adverse outcomes, is also a proxy for fetal movement counting and thus part of the intervention under study – not a pregnancy outcome. Rather would the single parts in the compound measure indicate the effects of the intervention.

In 1989, Grant *et al*. [14] conducted a large controlled, multicentre, cluster randomized trial, comparing formal fetal movement counting vs. counting only for risk pregnancies in a total population. Grant *et al*. did not identify any significant reduction in the in rates of unexplained stillbirth for women using a counting chart. Several methodological issues have been identified that have raised questions about the validity of the results and conclusions [2], [41], [42]. However, in spite of these serious critics, the Grant study has had an exceptionally powerful effect, and has often been cited as evidence against the usefulness of fetal movement counting [2], [16], [18], [23], [42].

### Identification of fetal growth restriction

This study was underpowered to detect any difference in perinatal mortality rates. However, we identified improved identification of fetal growth restriction, which is the most frequently reported association to decreased fetal activity and adverse outcomes [6], [7], [20], [43]. Identification of fetal growth restriction is of great importance; as growth-restricted fetuses who are *undetected* antenatally have a higher mortality than those that are detected prior to delivery [44], [45]. The improved antenatal identification of growth-restricted fetuses may have facilitated improved monitoring and timing of delivery.

### Increased maternal awareness by formal fetal movement counting

The universal self-screening performed as pregnant women perceive decreased fetal movements, may be the first identification of fetal compromise [2], [5], [6]. In line with our previous studies [11], [12], women in the current study came to the hospital with their concern about decreased fetal activity earlier in the pregnancy. In spite of not reaching statistical significance due to small sample size for this specific purpose, this is an important clinical finding, as unsafe delay in maternal reporting of decreased fetal activity was reduced. Empowering pregnant women in this universal self-screening through a daily routine of monitoring fetal activity may improve her assessment and timely reporting of decreased fetal movements.

### Use of health care resources – examinations and interventions

The frequency of consultations because of maternal report of decreased fetal movements in the current study is approximating previous studies [2], [11], [46]. There is a concern among health professionals that fetal movement counting may induce time consuming and unnecessary investigations [14], [21], [22]. The current study has demonstrated that fetal movement counting does not induce more use of health resources; this is an important finding to disprove this concern.

### Pregnancy outcomes

One of the factors associated with decreased fetal activity is a low Apgar score [47], [48]. The clinical relevance of a low Apgar score is questioned [49], but regardless of the cause, it indicates fetal compromise and is as such an unwarranted outcome [50]. It has been have shown that infants with 1-minute Apgar scores ≤3 have an increased risk of later disability compared with infants with normal scores [38], [51]. In our previous quality improvement study we found a tendency towards lower rates of severe neonatal depression among pregnancies presenting with decreased fetal movements [11], [12]. This randomized trial adds to this finding by identifying lower rates of severely low Apgar scores in the intervention arm.

By using a clinically oriented definition of fetal growth restriction, including significant deviation from normal growth by serial ultrasound, an intervention that led to large proportions of pregnant women being examined by serial ultrasound would provide skewed results inflating detection rates. This was not the case in this study, as the use of ultrasound was identical in both arms of the trial. Although the consultation rates were similar between the groups, we found an improved identification of fetal growth restriction and perinatal outcome in the intervention group. Uniform information and systematic monitoring of fetal activity in this group may have improved the mothers' ability to distinguish normal variations in fetal activity from changes representing risk. Thus, the women contacting the delivery clinics with their concern for decreased fetal activity in the intervention group may have been better selected.

### Methodological considerations

The strength of this study lies in its experimental design. Furthermore, the sample size was sufficiently large to permit cautious generalization of the findings, although the primary outcomes are composed of few cases, and size of the effect must be interpreted with caution. The sample was representative for the total population of pregnant women in Norway with respect to demographic characteristics, although the lower proportions of smoking women in the study sample may indicate a bias towards healthier pregnancies. Subgroup analyses were not pre-specified in the protocol, as the actual groups are too small to have enough power to proclaim any secure effects. Therefore, subgroup analyses have not been performed, according to the CONSORT statement [52].

The population of pregnant Norwegian women is relatively homogeneous; the women who participated were predominantly employed, cohabiting, white, and well-educated; a typical Scandinavian population. Therefore, generalizations should be limited to similar populations.

It is impossible to restrict the information about fetal movement completely to only one part of a population. Pregnant women are frequent users of social networks, and share their personal experiences and views [53]. The recruitment brochure informed that the purpose of the study was to improve our knowledge about the effect of fetal movement counting, and as simple randomization procedure was chosen, friends and neighbors may have been in different allocation groups. The intervention group received additional information about fetal activity and how to register and interpret the fetal movement pattern, but this information may also have reached the women in the control group. This may have contributed to an increased awareness about fetal activity in the total sample, as well as among the health care providers. However, the aim with this study was to evaluate the effects of increased awareness to fetal activity by performing a regular and formal fetal movement counting procedure. Thus, the potential increased awareness towards fetal activity in the total population during the study period, does not affect the validity of the results.

Although, all women must have heard about fetal movement counting in the recruitment process, only 143 (30.2%) reported that they knew about fetal movement counting when asked directly in a questionnaire. Of the women who knew about fetal movement counting, 62 (43.4%) had heard or read about it in the information brochure, whereas 81 (56.6%) had obtained information from the Internet, friends, or their midwife or physician. However, only one woman in the control group (0.2%) had used a fetal movement chart, indicating a clear separation between the groups.

Knowledge about the assignment group could potentially affect the observation, examination or the intervention of the participants. However, there was no difference between the intervention and control groups with regard to the frequency of use of any kind of observation or examination or follow-up, whether in the total sample or among the women reporting decreased fetal activity and such bias thus seems unlikely.

### Further research

The overall goal for fetal movement counting is reduced fetal mortality. As the perinatal mortality rates are as low as 4.4/1000 in our population [26], there is a need for a large, multi-centre, randomized, controlled trial in a more heterogeneous population than ours to investigate a broad spectrum of the effects of fetal movement counting on mortality rates. The potential benefit of empowering women's self-screening abilities may be significant in a wide range of populations.

Regardless of the debate on the effect of fetal movement counting, in current antenatal care, monitoring fetal activity is ongoing, largely as an unstructured self-screening procedure, administered and interpreted by the pregnant women individually [19], [21]. As the maternal perception of decreased fetal activity is the most important screening tool for fetal compromise [2], [9]–[11], this screening neither should, nor can be stopped – only improved. Implementation of fetal movement counting would not introduce a new screening, but only attempt to improve the value of the existing maternal self-screening.

### Conclusions

Maternal ability to detect clinically important changes in fetal activity seemed to be improved by fetal movement counting; there was an increased identification of fetal growth restriction and improved perinatal outcomes, without inducing either more frequent consultations at hospitals or increased frequency of interventions before or during delivery. Further research is needed to assess the effects of fetal movement counting on hard outcomes such as stillbirth rates.

## Supporting Information

Checklist S1 CONSORT Checklist.
(DOC)

Protocol S1 Trial Protocol.
(DOC)
