# Supplementary material for: Fetal Movement Counting Improved Identification of Fetal Growth Restriction and Perinatal Outcomes – a Multi-Centre, Randomized, Controlled Trial
Source: PLoS One. 2011 Dec 21;6(12):e28482. doi: 10.1371/journal.pone.0028482 (PMC3244397; doi:10.1371/journal.pone.0028482)
Supplement: Protocol S1 — Trial Protocol. (DOC) [file pone.0028482.s002.doc]

**Effects of fetal movement counting – a multi-centre randomized controlled trial**

**Project description**

**Oslo, March 29, 2007**

Eli Saastad

RN, RM, MSc, PhD student at the Medical Faculty, University of Oslo and Akershus University College

[1 The Femina collaboration and projects 4](#__RefHeading___Toc162923622)

[2 Effects of fetal movement counting – a multi centre randomized controlled trial 5](#__RefHeading___Toc162923623)

[2.1 The current situation in the population to study 5](#__RefHeading___Toc162923624)

[2.2 Maternal ability to identify significant changes in fetal activity level 5](#__RefHeading___Toc162923625)

[2.3 Fetal movement counting – advantages and disadvantages 6](#__RefHeading___Toc162923626)

[2.4 Effects on the women’s feeling of well-being 7](#__RefHeading___Toc162923627)

[2.5 Maternal-fetal attachment 7](#__RefHeading___Toc162923628)

[3 Aim and outcome measures 8](#__RefHeading___Toc162923629)

[4 Methods of data collections 9](#__RefHeading___Toc162923630)

[4.1 Study design 9](#__RefHeading___Toc162923631)

[4.2 Sample 9](#__RefHeading___Toc162923632)

[4.3 Recruitment of responders and data collection 9](#__RefHeading___Toc162923633)

[4.3.1 Randomizing procedure 10](#__RefHeading___Toc162923634)

[4.3.2 Variables 10](#__RefHeading___Toc162923635)

[4.3.3 Psychometric Instruments 10](#__RefHeading___Toc162923636)

[4.3.3.1 Mother-child attachment 10](#__RefHeading___Toc162923637)

[4.3.3.2 Maternal mood state 11](#__RefHeading___Toc162923638)

[4.3.3.3 Self-efficacy 11](#__RefHeading___Toc162923639)

[4.3.3.4 Self-esteem 11](#__RefHeading___Toc162923640)

[4.3.3.5 Maternal well-being 11](#__RefHeading___Toc162923641)

[4.3.4 Perinatal factors 11](#__RefHeading___Toc162923642)

[4.3.4.1 Obstetric history 11](#__RefHeading___Toc162923643)

[4.3.4.2 Identification of perinatal risk factors in actual pregnancy 11](#__RefHeading___Toc162923644)

[4.3.4.3 Perinatal factors with relevance for maternal concern and mother-child attachment 12](#__RefHeading___Toc162923645)

[4.3.5 Demographic information 12](#__RefHeading___Toc162923646)

[4.3.6 Information about fetal movements 12](#__RefHeading___Toc162923647)

[4.3.7 Experiences with FMC 13](#__RefHeading___Toc162923648)

[4.3.8 Experiences if examined because of DFM 13](#__RefHeading___Toc162923649)

[4.4 Data analyses 13](#__RefHeading___Toc162923650)

[4.5 Overview over data collection 13](#__RefHeading___Toc162923651)

[5 Time schedule 13](#__RefHeading___Toc162923652)

[6 Ethics and applications 14](#__RefHeading___Toc162923653)

[7 Budget for the RCT 14](#__RefHeading___Toc162923654)

[8 Power analyses 14](#__RefHeading___Toc162923655)

[8.1 Identification of risk pregnancies 14](#__RefHeading___Toc162923656)

[8.2 Maternal concern 15](#__RefHeading___Toc162923657)

[8.3 Maternal-fetal attachment 15](#__RefHeading___Toc162923658)

[9 Appendix I: Flowchart data collection 16](#__RefHeading___Toc162923659)

[10 References 17](#__RefHeading___Toc162923660)

# The Femina collaboration and projects

The Fetal Movement Intervention Assessment (Femina) collaboration engages several researchers worldwide with different aims and approaches.

Fetal activity serves as an indirect measure of central nervous system integrity and function, and regular FM can be regarded as an expression of fetal well-being^1;2^. Pregnancies in which the mother report decreased fetal movements (DFM) may indicate danger for the fetus^2;3^, and is associated with adverse outcomes as fetal hypoxia, growth restriction, preterm birth and stillbirth^4-7^. The unborn child responds automatically to hypoxia by redistributing blood flow away from the non-essential organs^8^ and reduction of non-vital activities^9^; *movements*. DFM is experienced by 4-15% of all pregnancies^3;4^.

Femina started in May 2004, with registration of the women seeking to 14 Norwegian hospitals for their worry because of DFM. Another 14 hospitals are participating in the UK, USA, Australia and New Zealand. The overall aim is to improve pregnancy health, pregnancy outcomes and child health through better understanding, awareness and management of fetal activity in general and DFM in particular. This include improving knowledge and clinical management through 1) Learning their epidemiology and outcome, 2) Improving the quality of care by health professionals, 3) Improving maternal information and vigilance, 4) Exploring the basic associations between fetal activity and outcome, 5) Testing new and improved approaches to fetal movement counting and 6) studying fetal growth and placental pathology in pregnancies with DFM.

The literature describes inconsistent and overmedicalized policies regarding the identification, assessment and monitoring of DFM^3;10;11^. There is a lack of evidence based knowledge of “normal” vs. “abnormal” fetal movements. Apparent “limits of normality” are mostly based on high risk pregnancies and concepts of DFM^4;12^. Femina has documented a wide spread of the health professionals’ view on FM’s relevance and significance as a marker for fetal well-being^11^, and there are large variations in management when women present their concern because of decreased fetal movements, how, and to what extent these risk pregnancies are identified in the population^3^. None of the hospitals involved in Femina in Norway had written guidelines before Femina started. They have however, responded to this sub-standard situation by consensus-driven quality-improvement projects starting in November 2005 on the two issues that need to follow each other – information to women and in-hospital management.

Femina has prospectively collected a population-based cohort of more than 5500 cases from about 82 000 pregnancies. Preliminary results from Femina have shown significant possibilities for clinical quality improvements with regard to *management* in pregnancies with DFM and *maternal perception* to fetal movements. Our results show that most mothers in our population did interpret their baby’s FM as a sign of good health, but that what they considered a reason for concern varied greatly – ranging from concerns because their baby kicked less than their friends baby (17%), to not contacting health professional even with total absence of FM (unpublished), and the few that actually contacted health professionals frequently receive non-productive or even dangerous information, ranging from telling the mother that she should feel 25 kicks/hour, to “reassurance” that it is only DFM after total absence of FM for over 24 hours^11^. In our Norwegian population, 50% waited more than 24 hours without any FM before they contacted health professionals. They did not understand the urgency and severity of this sign. When finally contacting help, they received variable help, as management consumed considerable health resources without any guidelines or monitoring of outcomes^3;11^. With lacking information to health professionals and pregnant women, as much as 50% of women affected by stillbirth perceive a reduction of FM, in many cases for several days before death^4;5;13-15^.

The current situation in the field of DFM is now a vast lack of basic knowledge and great opportunities for improvement and prevention both through maternal vigilance and in professional management. This study is a part of Femina, and includes the mothers’ perception of fetal movements and their management if experiencing DFM. Principal investigator for the Femina collaboration is J. Frederik Frøen, MD, PhD, researcher at the Norwegian Institute of Public Health. He will be the principal supervisor for my PhD. Co-supervisor is Tone Ahlborg, RN, RM, PhD at Akershus University College.

# Effects of fetal movement counting – a multi centre randomized controlled trial

## The current situation in the population to study

Before the studies included in Femina, there have been no randomized controlled trials of any aspects of management of DFM or on effective information to pregnant women. This situation yields unique opportunities to study the significance of DFM in depth, and also to approach one of the more controversial issues in antenatal care; the formal use of kick charts for total populations. Several case-control studies and one small Danish randomized controlled trial have indicated that the use of kick charts could significantly reduce stillbirths^16^. The only large randomized controlled trial compared kick charts for all women versus kick charts for all risk pregnancies in the population^17^. While the stillbirth rate in their population fell significantly during the study period with high vigilance and awareness among both pregnant women and health professionals (despite the failing management of DFM where 10% died in hospital due to clinical error), they failed to show significant differences between the groups.

There is little controversy in that pregnant women should be informed and guided to understand the significance of significantly decreased or absent fetal movements, and little controversy that they should receive a basic evaluation when presenting with symptoms. Yet the question remains – should all women use a formal kick chart? Does it really represent a helpful tool to increase their vigilance or their ability to distinguish pathology from normal variation? Does it promote maternal-fetal attachment? Is it harmful? Is it stressful? Does it cause an unneeded increase in the use of health services, or does it help provide better care to those who need it?

## Maternal ability to identify significant changes in fetal activity level

Maternal perception of FM is influenced by several factors; gestational age, type of fetal movement, maternal position, obesity, parity, placental location and psychological factors^18^. Several reports have stated that subjective registrations and assessments of decreased fetal activity might be useful in preceding many pregnancies with adverse outcomes^2;4;19;20^. Valentin has described a great interindividual variability in fetal activity and a small intraindividual variability^12^ and the most important current identifier of DFM is the women’s perception of what is a decrease of FM^12^. DFM reported by the mother in the week prior to delivery, has showed to be a statistically significant prognosis of fetal wellbeing, with a sensitivity of 57.7% and 56.5% for predicting alterations in fetal tests in normal and pathologic pregnancies and a specificity of 96.2% and 88.0% for normal and pathologic pregnancies respectively^20^.

The medicalization of perinatal care may have affected pregnant women’s view on own ability to recognize warning signals. However, the past several years, it seems to be an ongoing process against the medicalized perinatal care, and governmental reports in several countries have advised that women should be enabled to make and implement informed choices and the mothers have been increasingly encouraged to take an active part in making decisions regarding their care. In line with guidelines for antenatal care from WHO^21^ and NICE^22^, the new Norwegian guidelines for standard antenatal care also focus on demedicalization of pregnancy; with reduced frequency of standard antenatal controls and less tests^23^. Demedicalization in antenatal care implies to give the power back to the women, stimulate her listening to her body’s signals and improve the trust in non-instrumental signals. However, there seems to be a “missing link” – women are recommended fewer standard controls, but *without* receiving information and tools to be left with this responsibility. The mothers have not given relevant tools to be able to take care for their unborn child. This may affect their reporting of DFM and consequently time-consuming and frequently unnecessary investigations. We want to develop scientific knowledge on the pregnant women’s perception of FM and interpretation of significant changes in the FM pattern. Information and FMC could be a tool for getting to know their unborn child better, give the mothers a greater sense of control of her pregnancy and improve their ability to act on signs of complications at an optimal point of time.

The basic principle for antenatal care is to identify risk conditions where further observation or intervention is indicated in order to improve health for the mother or the child. An intervention may therefore be a positive action, e.g. if a CS is performed after a mother has experienced DFM and the child was in a threatened condition. Therefore, this study aims will be the number of identified risk pregnancies, not the end point of the pregnancies.

## Fetal movement counting – advantages and disadvantages

Maternal perception of fetal movements has, over the years, become recognized as a valuable tool for early detection of fetal compromise. The rationale for fetal movement counting is that adverse pregnancy outcome can be prevented by acting immediately when the woman reports decreased fetal movements. However, the importance of FMC in assessing fetal well-being is controversial. FMC is simple and can be done at home. It is economical, as there are no human or material resources needed, but it does intrude on the woman’s time. FMC might reduce fetal death and asphyxia by precipitating timely intervention, but on the other hand, it might increase obstetric interventions and prematurity. It is important to establish whether in practice benefits outweighs risks or vice versa, both as a routine procedure and in selected high-risk pregnancies, confirmed in a Cochrane review. This review conclude that there is a lack of knowledge about the sensitivity and specificity of FMC; its effectiveness in decreasing the perinatal mortality in high-risk and low-risk women; its acceptability to women; how easy it is for women; and the best fetal movement counting method^24^.

Several methods for monitoring fetal movements have been used; the two most common are “daily movement count”^19^ or “count-to-ten”^25^, the latter has been found to be most user-friendly^26;27^. The Femina collaboration has chosen a modified “count-to-ten”-method; the mothers are asked to note the time it takes to feel 10 movements after she have felt one movement; counting will start when the baby is awake. This method reduces the normal variations, since the counting period not will start when the baby is sleeping. With a focused counting according to this method, the average pregnant woman in the third trimester will perceive 10 FM within 20 minutes, but only after two hours it is a rare event^11;28;29^.

## Effects on the women’s feeling of well-being

Psychological changes as a result of attention to fetal activity could be negative or positive. FMC might increase anxiety or might be reassuring. Studies evaluating use of FMC, have shown that FMC raised maternal anxiety levels^17;30^, but others did not find any association with anxiety^31-33^. Even if Grant & al^17^ found FMC as having a little, if any, stress inducing effects on women, they suggested that any maternal anxiety experienced is a reflection of more general concern about FM rather than concern prompted by formal counting. They also argued that stress and anxiety were significantly reduced when feedback of information was given to women regarding the health of their baby. Information and instructions are essential in clinical use of FMC^12^, and communication is seen as vital between the women and the providers of antenatal care for anxiety to be successfully allayed in women using a fetal movement counting policy. The Grant-study showed that FMC gave the mothers a non-significant increase in confidence and control^17^. Mikhail & al found better attachment between mother and child and slightly decrease in feelings of wellbeing for those who counted^34^.

Fetal movement counting itself will not reduce the likelihood of adverse pregnancy outcome. This will happen only if both women and caregivers respond adequately to the signals of decreased fetal activity. More observant women are not equal with more concerned women. In many cases, an adequate level of concern may also be warranted, versus a lack of vigilance putting their pregnancy at risk. Maternal individual responsibility is desirable. FMC may have positive effects on recognizing pregnancies at risk. However, it can also induce mental non-wellbeing, like anxiety, stress, irritation, tiredness, feeling guilty if not practicing FMC, or it may intrude in other activities for the mother or in other ways affect her quality of life. These aspects remain unknown and will be one of the aims of this study.

## Maternal-fetal attachment

The concept of maternal-fetal attachment (MFA) has been used in different ways with different theoretical frameworks to describe the relationship between a pregnant woman and her fetus. The research on prenatal attachment is characterized by low validity, including inadequate operational definitions of the construct, small, homogenous samples, and a lack of sensitivity to cultural issues^35^. It is a lack of knowledge on the processes by which MFA develops, including physiological and psychological mechanisms that could shape the development of MFA, and there is a lack of knowledge on what constitutes “normative” scores^36^, even if Condon in two old studies has estimated that about 8 to 15 per cent of women develop minimal attachment to their child^37;38^.

Maternal feelings and sensitivity to her child develop along a continuum throughout pregnancy as a result of dynamic psychological and physiological events^39^. A broad spectrum of MFA has been observed during pregnancy. The rate and degree of MFA development appears to be influenced by gestational age at quickening, amount of fetal movements, pregnancy history and the mother’s own attachment history. Fetal movements, ultrasound images, the delivery, the baby’s first smiles, are all opportune moments to consolidate the desire for the infant^40^. Development of MFA is heightened by fetal movements^41^. The development of MFA is rooted in the desire to have a baby. An unplanned pregnancy or an unwanted baby may compromise the parent-infant attachment^42^. It is assumed that the MFA is relatively stable when the women have reached their individual level, this is reported to develop in particular in the second trimester^43^.

Attachment theories state that the affectional tie between a mother and her infant is essential for enhancing the child’s early survival and later capacity for getting along with others^34^. Associations between prenatal and postnatal attachment have been anticipated through many years, but there have been inconsistent findings; some studies have failed to report any significant association between prenatal and postnatal attachment^35;39^, others have identified associations between pre- and postnatal attachment^44^. Maternal prenatal attachment during the third trimester of pregnancy is associated with the postnatal maternal involvement, and can serve as an important diagnostic aid in identifying those women for whom the mother-child interaction is likely to be sub-optimal^45^. Women intending to breast feed had higher level of maternal fetal attachment^46^. It has been assumed that a healthy attachment between mother and the unborn babies is supposed to continue to be a good, sensitive interaction postnatally^43^. A child’s secure attachment to the mother is positively correlated with the child’s exploration ability, problem-solving ability, curiosity and control in the preschool years^47^ and strong prenatal attachment may decrease the risk of child abuse^48^.

Maternal mood state has been consistently related to ratings of MFA^49^; prenatal depression had a negative relationship to MFA, and MFA a positive relationship with positive prenatal health practices of the pregnant woman that may affect her health, the health of the fetus or the pregnancy outcome, like diet, sleep, exercise, abstaining from harmful substances, such as alcohol, cigarettes or drugs and prenatal care. However; positive MFA may mediate the negative effects of depression on positive health practices^49^. The technological development in western nations over the past 30 to 40 years has changed conceptions about pregnancies and the fetus. Women can detect pregnancy earlier and are able to view high-resolution images of their fetus at earlier dates. This knowledge may serve to allow women to adopt optimal health practice earlier.

Inconsistent findings on factors affecting the MFA are the reason for including elements of self-efficacy and self-esteem in the surveys. General self-efficacy is the belief in one’s competence to cope with a broad range of stressful or challenging demands^50^. Self-esteem is regarded as a stable personal trait^51^. Identification of low self-efficacy and self-esteem prior to FMC is important to assess possible interactions with MFA.

Since fetal movements is found to be the factor that increase the MFA, FMC be a tool for the mothers that might increase or decrease the their positive feelings toward the pregnancy and infant and stimulate to increased attachment between mother and child.

# Aim and outcome measures

The aim is to test effects of using formal kick counting chart in the third trimester of pregnancy in an unselected population. The research questions are:

Does Fetal Movement Counting:

1. Improve the identification of risk pregnancies / pathology?

*Primary outcome measures*: Numbers of identified pathological conditions in pregnancies (fetal growth restriction (FGR), acute caesarean section on fetal indication/non–reactive non-stress test (NST), oligohydramnios, pathologic blood flow in arteria umbilicalis, maternal perception of absent fetal movements for more than 24 hours before admission to hospital, or perinatal death).

*Secondary outcome measures*: Frequency of consultations because of maternal concern, use of resources in evaluation of these pregnancies (NST, ultrasound, Doppler, recurrent consultations), induced or spontaneous start of delivery, mode of birth.

1. Affect the women’s well-being?

*Primary outcome measure*: Maternal concern.

*Secondary outcome measure:* Maternal satisfaction with use of FMC and sense of control in interpretation of signals from own body and child.

1. Stimulate maternal-fetal attachment antepartum?

*Primary outcome measure*: Maternal-fetal attachment.

*Secondary outcome measure:* Health promoting behavior in pregnancy (smoking habits, use of alcohol, attending standard program for antenatal care).

# Methods of data collections

## Study design

Randomized controlled trial with women which respectively will:

a) Be given written information about fetal activity and instructions how to use and interpret FMC-charts – the intervention group

b) Follow standard antenatal care according to the Norwegian Guidelines – the control group

## Sample

A total population of singleton, Norwegian-speaking women will be invited after screening ultrasound in pregnancy week 17, exclusive pregnancies with severe anomalies or other cause to consider termination or non-Norwegian speaking/understanding women. Women will be recruited from hospitals not included in existing Femina collaboration. The period of inclusion should be intensive and as short as possible and in a geographical spread area, in order to minimize the potential contamination of information between participants, and to make the conditions for both health professionals and respondents as homogenous as possible.

Sample power analyses based on the preliminary observational study is done to estimate the number of respondents needed. The preliminary estimate with 80% power and a significance level of 0.05, is a total of 538 pregnancies in each arm of the trial. This will be further assessed after evaluation of the first part of the inclusion period.

## Recruitment of responders and data collection

Women will be recruited from the hospitals’ lists over women having been at the ultrasound screening in pregnancy week 17-19. A flow chart over data collection is appended (Appendix 1). All women will receive a letter in pregnancy week 23-24, asking to participate in a study of attitudes during pregnancy with a random possibility to be asked to perform FMC in the third trimester. Mothers, who agree to participate, do this by signing an informed consent and completing a baseline questionnaire (QI). On completion of the baseline inventories, but before inspection of the results, an assistant will perform the randomization into the FMC-group and the control group.

Women in the FMC-group will be asked to count fetal movements daily from gestational week 28. These women will be telephoned one week after commencement, to discuss questions or problems with the charting technique. Women in the control group will be told about the next survey at 34 weeks of gestation. Women in both groups will be asked to perform prospective, structured registrations – like a diary - if they were concerned about DFM and return these diaries to the study group after the delivery.

The questionnaires will be distributed in pregnancy weeks 24 (questionnaire I) and 34 (II). Answering the questionnaires can be done either by paper or by web-pages with interactive questions. An appropriate web-solution will be used for electronic data collection. Alternative retrieval for paper version: Scanning of questionnaires at the NIPH.

### Randomizing procedure

Randomization will be determined by computer-generated random allocation. The allocation of the responders will be blinded for the researchers and the recruiting midwife.

### Variables

The questionnaires will be developed to respond to the cited research questions in cooperation with a resource group for Femina; consisting of midwives, GP’s, obstetricians and pregnant women. When available, validated self-report psychometric scales are chosen.

### Psychometric Instruments

Where no Norwegian instruments were available, the English inventories (PAI and CWS), have been translated for this study into Norwegian by one of the researchers and back translated by another person, both fluent in English.

#### Mother-child attachment

Reviewing the literature, most studies measure MFA by the Maternal-Fetal Attachment scale (MFAS)^39^, less frequently used are Müller’s Prenatal Attachment Inventory (PAI)^48^ and Condon’s Maternal-Antenatal-Attachment Scale (MAAS)^38^. MFA as measured by these scales is consistently related to pregnancy planning, strength of the marital relationship, gestational age and maternal depressed mood^41^. The variables of maternal age, parity, self-esteem and socioeconomic status are inconsistently related to MFA across studies (Erikson 1996). Social support of family members and peers is a significant predictor of MFA; perceived support of prenatal care providers was correlated with MFA at 0.74, providing further evidence that psychosocial support is a critical component of prenatal care^52^. In spite of different framework in these three inventories, it has been shown correlation between the MFAS and PAI (r=0.41, p<0.001)^44^, and Condon’s scale has been assessed to be very similar to those reported by investigators using the MFAS^42^. The PAI is assessed to be the most suitable tool for measuring MFA and is chosen in this study; this inventory emphasizes affiliation rather than behavior and the psychometric properties remain quite adequate in the PAI, with high construct validity and reliability, total score Cronbach’s alpha was reported to be 0.87^49;53^. This instrument is designed to measure, from the mother’s perspective, the affectionate relationship that develops between a mother and her unborn baby and not merely the experience of pregnancy as a physical state or her appreciation of the tasks concerning the motherhood role^48^. The PAI is a 21-item inventory is rated on a 4-point Likert-type scale indicating how often the mother has affectionate thoughts or behaves affectionately toward the fetus. Individual items are summed and treated as ratio data. Higher scores indicate greater attachment. Measurements with the three inventories have mostly been performed in pregnancy week 33-36.

#### Maternal mood state

MFA is consistently related to maternal depressed mood^41^. Identification of depression prior to FMC is important to assess possible interactions with MFA. The 25-item Hopkins Symptom Checklist (HSCL-25) is a self-reporting inventory to obtain information on symptoms on emotional distress in normal populations^54^. A short version (SCL-8) has been developed at The Norwegian Institute of Public Health (unpublished), and contains for items for depression and four for anxiety. Both versions have 4-point Likert scales.

#### Self-efficacy

GSE (Generalised Self-Efficacy)^55^, has been shortened into a five-item scale and translated into Norwegian^56^ and includes 4-point Likert scales. GSE appears to be a universal construct that yields meaningful relations with other psychological constructs.

#### Self-esteem

The Rosenberg Self-Esteem scale(RSES)^57^ has been validated and translated into Norwegian and is recommended by the Norwegian Registry of Psychometric Scales^51^. On basis of recommendations here and in line with the Norwegian Mother and Child Cohort study^58^ a four-item scale of the RSES is chosen with 4-point Likert scales.

#### Maternal well-being

The Cambridge Worry Scale (CWS)^59^ has demonstrated good reliability and validity^60^, also in a Scandinavian population^61^. The measure contains 16 items, measuring women’s major worries during pregnancy in a four-factor structure; socio-medical, own health, socio-economic and relational aspects. Responses are made on a 6-point Likert-type scale (from 0=”not a worry” to 5=“extremely worried”). An open-ended question at the end allows the respondents to tell about other worries not in the list.

### Perinatal factors

#### Obstetric history

Obstetric history and pregnancy outcome will be obtained from copies of case notes. Questions about these issues and factors associated with MFA will be included in the surveys as covariates according to the overview.

#### Identification of perinatal risk factors in actual pregnancy

This study is not suited to use primary outcome measures which are frequent used in traditional epidemiological research on these topics. Earlier studies on research on fetal movements and the relevance and significance of FMC, consequently use primary outcomes as perinatal death, fetal growth restriction, severe fetal asphyxia, acute caesarean section, preterm birth and transfer to NICU. However, these “hard” and relatively rare outcomes are not useful for this study, since this study aims to register the mothers’ ability to identify significant changes of fetal activity, i.e. the number of identified pregnancies with a risk condition on basis of the mothers’ interpretation of fetal movements and management if significant changes. Therefore, the primary outcomes in this study are identification of risk pregnancies, e.g. number of consultations where an intervention or further observation was indicated on basis of the mothers’ perception of DFM.

Primary outcome measures – pathology identified according to Norwegian guidelines:

- Fetal growth restriction (FGR) identified by ultrasound prior to birth:
- FGR at birth, unidentified prior to birth
- Acute caesarean section on fetal indication (exclusive protracted delivery)
- Non–reactive non-stress test (NST)
- Oligohydramnios
- Pathologic flow in arteria umbilicalis
- Maternal perception of absent fetal movements for more than 24 hours before admission to hospital:
- Perinatal death

Secondary outcome measures:

- Frequency of consultations because of maternal concern
- Use of resources in evaluation of these pregnancies (NST, ultrasound, Doppler, recurrent consultations)
- Induced or spontaneous start of delivery
- Mode of birth

#### Perinatal factors with relevance for maternal concern and mother-child attachment

The following elements will be reviewed:

- Planned pregnancy
- Infertility
- Previous pregnancies
- Due date
- Time for quickening
- Known fetal gender before birth
- Numbers of ultrasounds in this pregnancy

### Demographic information

Maternal age, weight, height, educational level, marital status, smoking habits and country of origin will be obtained from copy of the standard form for antenatal care (“Helsekort for gravide”).

### Information about fetal movements

This enables us to evaluate maternal experiences with the information about fetal movements and the subjective user-friendliness and utility value of the information. No measurement scales exist, and questions are made for this study. A four-point Likert scale ranging from 1 (do not agree at all) to 4 (completely agree) will be used.

### Experiences with FMC

No measurement scales exist to evaluate the users’ satisfaction with FMC. Questions were made for this study, including perceived stress, irritation, tiredness with practicing FMC, feeling guilty if not practicing FMC, and if FMC intrudes in other activities for the mother or in other ways affect her quality of life. A four-point Likert scale ranging from 1 (do not agree at all) to 4 (completely agree) will be used.

### Experiences if examined because of DFM

Maternal evaluation of quality of the encounter with a midwife or a doctor if examined because of concern will give important information on the women’s experiences of being given power from health professionals in her assessments of fetal activity. Questions were made for this study. Patient’s perceptions of quality of care are considered in two dimensions; medical-technical competence of the caregivers and the attitudes and actions of the caregivers. A four-point Likert scale ranging from 1 (do not agree at all) to 4 (completely agree) will be used.

## Data analyses

Data handling will be performed in SPSS 14.0. Best suited statistical methods will be chosen in the analyses. Participants will be handled according to the intention-to-treat-principle.

## Overview over data collection

|  | Survey I | Survey II | Copy of case notes |
| --- | --- | --- | --- |
| Maternal-fetal attachment |  | x |  |
| Mental well-being | x | x |  |
| Maternal depression level | x |  |  |
| Self-efficacy | x |  |  |
| Self-esteem | x |  |  |
| Experiences with FMC to the intervention group |  | x |  |
| Experiences if examined because of DFM |  | x |  |
| Demographic details | x |  | x |
| Obstetric history |  |  | x |
| Planned pregnancy | x |  |  |
| Number of US’s in this pregnancy |  | x |  |
| Known fetal gender | x | x |  |

# Time schedule

| **Data-collection/focus** | **Started/estimated started** | **Estimated to be finished** |
| --- | --- | --- |
| Preparation of the studies | Sept 06 | April 07 |
| RCT: information versus fetal movement counting | Recruiting respondents: May 07 | December 07 |
|  | Data analyses: January 08 | January 09 |
| Publication of four scientific articles |  |  |
| 1) Maternal perception of fetal movements in a total population – two cross-sectional surveys |  | June 07 |
| 2) FMC and identification of perinatal risk factors |  | March 08 |
| 3) FMC and maternal concern |  | October 08 |
| 4) FMC and maternal-fetal attachment |  | January 09 |

# Ethics and applications

The Femina-study and “Tell Trivselen” are approved by the Regional Committee for Medical Research Ethics. Femina is advised by The Norwegian Data Inspectorate and “Tell Trivselen” was advised by The Norwegian Data Inspectorate in June 2006. The study will be applied for accept by the Regional Committee for Medical Research Ethics, The Norwegian Data Inspectorate and Sosial- og Helsedirektoratet, and will be reported to the database for clinical trials ([www.clinicaltrials.gov](http://www.clinicaltrials.gov/) ) before start.

All participants will be invited to contact the study group by telephone or by mail. This will ensure that anxious and worried women could get advices about how to get appropriate help if needed. According to this, participants in the intervention group will be contacted by telephone one week after FMC start. This is to increase the compliance to the counting and to ensure correct interpretation of the instructions about the counting method.

# Budget for the RCT

| PhD scholarship (75% time) | NOK 1.456.000 |
| --- | --- |
| Operating expenses | NOK 100.000 |
| Payment for recruiting midwifes | NOK 648.000 |
| Printing of questionnaires | NOK 120.000 |
| IT consultant for interactive questionnaires | NOK 40.000 |
| Total | NOK 2.364.000 |

# Power analyses

Estimations of sample size needed to preserve the needed power vary widely with the issue being studied. Some examples on how effects of sample size will affect some of the measurements in this study are done by evaluating the effect of the intervention. This is done with the computer program PS Power and Sample Sizes^62^. The effects of changes is estimated by relative risk for dichotomous variables and means with standard deviations for continuous variables Power analyses only for the three primary outcomes are included here.

## Identification of risk pregnancies

The goal is to increase the accuracy of maternal perception of DFM as a predictor for need of examination or intervention. This will be measured by a compound measure of rates of identification of risk pregnancies related to DFM. Prevalence of risk conditions are estimated from preliminary results of Femina (unpublished) or the Norwegian Birth Register.

| Condition | Prevalence |
| --- | --- |
| Fetal distress: Acute caesarean section on fetal indication (excl protracted birth), non-reactive NST, pathological levels of fetal scap-pH or Apgar_5 min_ ≤ 3 | 8 % |
| Fetal growth restriction < 2.5 centile or pathological Doppler flow in arteria umbilicalis | 5 % |
| Absent fetal movements for more than 24 hours | 0.5 % |
| Intrauterine death | 0.02 % |
| Estimated prevalence of compound measure | 13.52 % |

The goal is a 10% increase of identification of these risk pregnancies, which gives an estimated sample of 538 in each arm of the trial with 80% power and a significance level of 0.05.

## Maternal concern

The goal is to identify no increase in the level of concern in the intervention group. In the original study with 1207 respondents in the 16 items (0-5) inventory with a 6-point Likert scale, unweighted mean scores across items and standard deviations for the survey in pregnancy week 35 were 1.13 (0.65)^59^. Detectable changes are estimated to be a change in mean of 0.12, which give an estimated sample of 462 participants in each arm of the trial with 80% power and a significance level of 0.05.

## Maternal-fetal attachment

Goal: To observe an increase the maternal-fetal attachment in the intervention group when compared to the control group. In the study with the best methodological quality^53^, mean score and standard deviation for MFA in pregnancy week 33 is 63.7 (10.2). Estimated detectable change is five within the groups, which gives an estimated sample of 64 participants in each group (power 80%, significance level 0.05). This means that this study is overdimensioned for the purpose to observe changes in the level of MFA.

# Appendix I: Flowchart data collection

Screening ultrasound

Excluded, mothers:

with multiple pregnancy

with fetal malformations and termination of pregnancy is considered

who not speak/understand Norwegian

Pregnancy week 17-18

Control group – standard antenatal care

Pregnancy week 28

Intervention group starts FMC

Telephone contact

Pregnancy week 29

Pregnancy week 34

Information about the study, invitation to participate and a form for informed consent to fill in

Questionnaire 34-K

Simple randomization

Post partum

Return of the counting chart

Questionnaire 34-I

Pregnancy week 22

Copy of case notes from hospitals from both groups

Questionnaire 22-F

# References
